# Effects of molecular interactions between the exposome and oxylipin metabolism on healthspan

**DOI:** 10.3389/fphys.2025.1584195

**Published:** 2025-07-01

**Authors:** Jennifer Hinman, Jamie K. Alan, Kin Sing Stephen Lee

**Affiliations:** ^1^ Department of Chemistry, College of Natural Science, Michigan State University, East Lansing, MI, United States; ^2^ Institute for Integrative Toxicology, Michigan State University, East Lansing, MI, United States; ^3^ Department of Pharmacology and Toxicology, East Leansing, MI, United States

**Keywords:** senescence, molecular mechanism, aging, oxylipin, metabolism, exposome

## Abstract

While aging is typically synonymous with the lifespan of an individual, the healthspan, or the total number of years an individual remains healthy and disease-free, is not necessarily related. A current, critical need in society has arisen as current populations are living longer than previous generations, thus increasing the number of people with age-associated diseases. However, the extent of all age mechanisms is not entirely known. Still, studies examining how the exposome, or an individual’s cumulative exposures throughout their life, can influence or modulate aging processes will strengthen our understanding of how to keep individuals healthy and disease-free longer, bridging the gap between lifespan and healthspan. In contrast, previous research has also demonstrated that the exposome impacts aging. One subcategory of the exposome, the specific external, encompasses daily exposures such as diet, lifestyle factors, and occupational and environmental exposures. In this review, we focus on the interactions between factors of the exposome, such as vitamins and minerals, and their effects on aging, cell death, cellular senescence, and unsaturated fatty acid metabolism. We also discuss the interaction between oxidative fatty acid metabolism and aging itself. Overall, understanding how various exposures impact both the oxylipin profile and healthy aging will inform future interventions aimed at improving the healthspan and quality of life for this large aging population.

## Graphical Abstract

**Figure:**
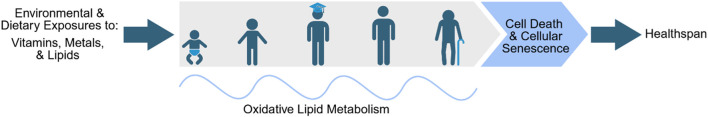
The goal of this review is to highlight the current understanding of the interactions between the exposome and oxylipin metabolism on healthy aging.

## 1 Introduction–aging

Aging is a natural, ubiquitous process that refers to the progressive deterioration of functions and regulatory processes, resulting in a reduced ability to maintain homeostasis when challenged by external stresses and increases the interindividual variability in physiological responses (Guo et al., 2022; Mangoni and Jackson, 2004). While the extent of all aging mechanisms is not entirely known, the current understanding of these complex mechanisms involves a combination of genetic, molecular, and environmental factors. Largely, aging is induced by the accumulation of damage due to exposure and response to a variety of stresses (Guo et al., 2022; Mangoni and Jackson, 2004). In the field of aging research, hallmarks of aging have been established including genomic instability, telomere attrition, epigenetic alterations, loss of proteostasis, deregulated nutrient-sensing, mitochondrial dysfunction, cellular senescence, stem cell exhaustion, and altered intracellular communication (López-Otín et al., 2013). As more insights on aging mechanisms have been uncovered, these hallmarks have been expanded upon to include disabled macroautophagy/compromised autophagy, chronic inflammation, and dysbiosis (Guo et al., 2022; López-Otín et al., 2023).

### 1.1 Increased lifespans in current populations

In the past 70 years alone, the human population has almost tripled, from 2.9 billion (1950) to 7.8 billion (2020) (Garmany et al., 2021). With continued societal and medical progress, the world population is expected to continue this steep increase in the coming decades. This increase in population is partially due to current generations living longer than previous generations with the average life expectancy increasing from 47 to 73 years of age in this same time span (Garmany et al., 2021). However, lifespan and healthspan, which is defined as the total number of years an individual remains healthy and disease-free, are not directly correlated. Decline in the healthspan and quality of life of elderly populations is largely due to age-associated diseases, leading to disability, frailty, and morbidity in these populations. Aging is a major risk factor and driver of a variety of health diseases and conditions affecting elderly populations (Guo et al., 2022; Hodes et al., 2016; Sierra, 2016). Specifically, aging has been shown to be a major risk in chronic diseases such as type 2 diabetes (Milanesi et al., 2000), cardiovascular disease (CVD) (Yan et al., 2021), chronic obstructive pulmonary disease (Mannino and Buist, 2007; Kukrety et al., 2018), osteoporosis (Chandra and Rajawat, 2021), osteoarthritis (Anderson and Loeser, 2010), Alzheimer’s disease (AD) (Hou et al., 2019; Cortes-Canteli and Iadecola, 2020), and Parkinson’s disease (PD) (Hou et al., 2019; Wood-Kaczmar et al., 2006). Thus, understanding the aging mechanism and how aging impacts healthspan will lead to novel interventions to improve the healthspan and the quality of life of this large aging population. Understanding the implications of perturbations to aging processes by the exposome, defined as an individual’s cumulative exposure throughout their life–can inform intervention methods to keep individuals healthy and disease-free longer, bridging the gap between lifespan and healthspan.

### 1.2 Current understanding of aging mechanisms, and the influence of the exposome and oxylipin profile on aging

Aging is a complex, multifaceted process that involves the interplay of various mechanisms. Multiple theories explaining the molecular mechanisms of aging have been postulated. Most of these theories can be separated into two main categories: programmed and damage or error theories. Programmed theories suggest that aging is driven by genetically programmed pathways that limit the lifespan based on changes in gene expression for maintenance, repair, and defense response systems (Jin, 2010). Underneath the overarching branch of programmed theories are the programmed longevity, endocrine, and immunological theories. However, damage or error theories suggest that aging is the result of damage accumulation (i.e., ROS, DNA mutations, protein misfolding) in cells and tissues from cumulative environmental exposures over time (Jin, 2010). Aging effects result from the decline in the body’s ability to repair this damage with age, leading to cellular dysfunction. However, one of the main characteristics of aging overall is the accumulation of cellular damage (Vijg and Campisi, 2008). Thus, many of the interactions of the aging hallmarks, cellular damage, and oxidative stress are summarized in Table 1.

**TABLE 1:** Aging hallmarks in oxidative stress and cellular damage.

Aging hallmark	Aging involvement *in vivo* changes	Citations
mitochondrial dysfunction	•Mitochondria are key components involved in oxidative stress regulation and a link between mitochondrial dysfunction and aging has been proposed •Mitochondrial DNA (mtDNA) lacks the repair mechanism of nuclear DNA making mtDNA more susceptible to mutations, and the accumulation of mtDNA mutations can cause mitochondrial dysfunction •Disruption of mitochondrial fusion and fission also results in fragmented and dysfunctional mitochondria •Increased numbers of mutations in mtDNA have been observed in various tissues during aging •Research suggests that progressive mitochondrial dysfunction during aging results in higher production of reactive oxygen species (ROS), in which excessive ROS production from mitochondrial dysfunction damages lipids, DNA, and proteins, leading to cellular damage	Guo et al. (2022), López-Otín et al. (2013), López-Otín et al. (2023), Liu et al. (2020), Zapico et al. (2013)
loss of proteostasis	•With aging, the cellular machinery responsible for protein regulation becomes less efficient •Dysfunction of protein regulation mechanisms results in the accumulation of misfolded proteins and protein aggregates, which have been linked to age-associated neurodegenerative diseases like AD, PD, and Huntington’s disease •Epigenetic changes also occur during aging ◦ A decrease in DNA methylation and alterations in histone modifications (primarily methylation and acetylation) can occur ◦ Dysregulation of histone modifications has been linked to age-related diseases and longevity	Guo et al. (2022), Hipp et al. (2019), Mendelsohn et al. (2017), Wang et al. (2018)
chronic inflammation	•Inflammaging, or the chronic, low-grade inflammation that increases with aging, is a major driver of many age-related diseases •Both senescence-associated secretory phenotype (SASP) and immune system decline (immunosenescence) can increase inflammation	Franceschi et al. (2000), Teissier et al. (2022)
stem cell exhaustion	•During aging, there is a decline in the regenerative capacity of stem cells needed for tissue regeneration and repair, leaving cells more prone to senescence •The number and function of most somatic stem cells decrease with aging and the microenvironment where stem cells reside can become less supportive for stem cell maintenance and function with aging •Age-associated stem cell exhaustion impairs the ability of tissues to regenerate after injury	Brunet et al. (2023)
cellular senescence	•The secretion components of senescent cells (i.e., SASP) consists of pro-inflammatory and growth-stimulating proteins that are secreted into the microenvironment surrounding senescent cells that can modulate local and systemic biological activities, and may also disrupt tissue structure and function ◦ The collection of growth factors, proteases, cytokines, and other factors with potent autocrine and paracrine activities within the SASP can self-reinforce senescence or affect neighboring cells and tissues, contributing to chronic inflammation and tissue dysfunction •Senescent cells accumulate with aging and have been observed in a variety of age-related diseases, contributing to the aging process •Some components of the SASP are enriched in the plasma of individuals during aging and age-associated diseases •Additionally, senescent cells display an increase in oxidative stress, which has been suggested to be due to dysfunctional mitochondrial accumulation	Rodier and Campisi (2011), Wang et al. (2024), Červenka et al. (2021), Di Micco et al. (2021), Chapman et al. (2019), Campisi (2003), Tanaka et al. (2018)

Decades of research have demonstrated that aging is impacted by the exposome. Exposome is broadly defined by environmental exposures which people face in everyday life. For example, a recent epidemiological study by Argentieri et al. conducted an exposome-wide analysis of all-cause mortality using the UK Biobank data and identified that 25 specific exposures are associated with proteomic aging (Argentieri et al., 2025). While specific mechanisms on how the exposome impacts aging remain understudied, research has shown that the exposome, including diet, modulates endogenous unsaturated fatty acid metabolism, which generates hundreds, if not thousands, of lipid metabolites (Liu et al., 2017; Rodrigues et al., 2022). These unsaturated fatty acid metabolites are key signaling molecules in many aspects of human physiology. For example, prostaglandins play an important role in inflammation, vascular tone, and pain perception (Innes and Calder, 2018). Recent studies also demonstrated that prostaglandins and the associated metabolic enzymes impact aging (Srour et al., 2024; Palla et al., 2021). However, the effects and associated mechanism of the exposome and unsaturated fatty acids metabolism on aging are underexplored. This review will discuss how senescence and aging pathways can be influenced by specific dietary and environmental exposures of the exposome (vitamins, metals, PUFAs, and oxylipins) and how the exposome can influence oxylipin (oxygenated polyunsaturated fatty acid derivative) metabolism. Overall, the review will examine how these exposures can impact healthy aging.

## 2 Exposome

**FIGURE 1:**
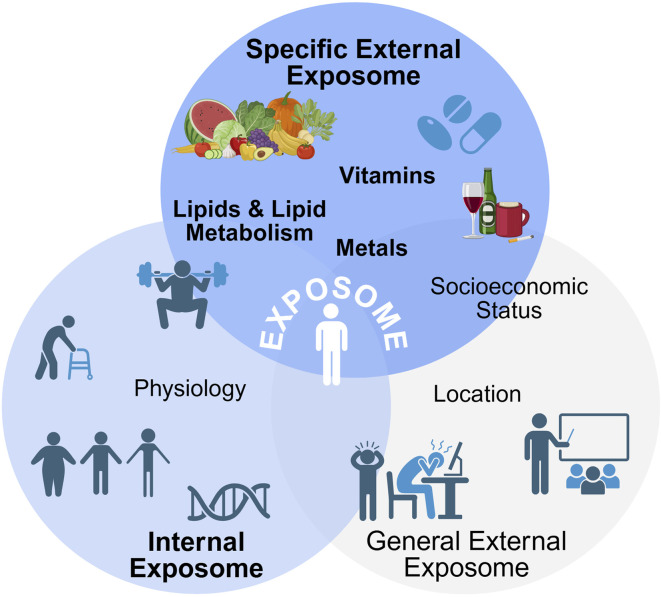
The complete exposome. The exposome can be divided into three subcategories–internal, general external, and specific external, with these subcategories overlapping and intertwining with each other. Overall, the exposome consists of total exposures an individual is exposed to in their lifetime. Created with BioRender.

The exposome (Figure 1) encapsulates all of the environmental (non-genetic) exposures experienced by an individual from an array of dietary, lifestyle, and demographic factors throughout the lifespan that impact health outcomes (Wild, 2005). Some researchers have broadened the definition of the exposome to include additional factors examining how the body reacts to environmental factors such as biological responses, behavior, and endogenous processes (Miller and Jones, 2014). The exposome includes a network of unique characteristics and exposures of an individual that may result in them being more or less susceptible to particular stressors in their environment (DeBord et al., 2016). Overall, the exposome can be broken into three subcategories: internal, specific external, and general external (DeBord et al., 2016; Wild, 2012). Despite the distinct exposure classifications, these subcategories are considered to be both overlapping and intertwining, and differentiating factors between these categories can be difficult (DeBord et al., 2016). While all three subcategories are vitally important in understanding how the exposome impacts human health and disease, this review will focus on the molecular interactions of specific external factors on aging and oxidative lipid metabolism. Thus, the internal and general external factors are out of scope for this review and have been extensively reviewed elsewhere. Here, we will discuss these briefly below and the corresponding reviews for specific exposomal effects on health have been included.

### 2.1 Internal

The internal or endogenous factors of the exposome encompass those that are specific to an individual. Specifically, these encompass the internal factors relating to host factors or the impact on cellular environments and processes. Internal factors can include physiology (Jin, 2010; Rappaport, 2011), aging (Guo et al., 2022; Mangoni and Jackson, 2004; Sierra, 2016; Vijg and Campisi, 2008; Fülöp et al., 2009; Fede et al., 2022; Tamburini et al., 2004), body morphology/somatotype (Carter and Heath, 1990; Koleva et al., 2002), physical activity (Thyfault and Bergouignan, 2020; Booth et al., 2012; Gorman et al., 2021; Grundy et al., 2004), and genome (Miller and Jones, 2014; Wild, 2012; Rappaport, 2011). These internal exposures can typically be measured using biomarkers of these endogenous processes.

### 2.2 General external

Despite overall exposures to the same environmental nuisances, individuals and groups can be impacted differently, and these differences can be partly attributed to the general external exposome. The general external factors are considered the social determinants of health (Friel and Marmot, 2011) as well as the wider effects of social, economic, and physiological status on health and disease (Wild, 2012). General external factors specifically consist of influences from location (Dummer, 2008; Institute of Medicine (US) Committee on Guidance for Designing a National Healthcare Disparities Report, 2002; Sogno et al., 2022; Dale et al., 2012), education level (Zajacova and Lawrence, 2018; Kitagawa and Hauser, 2013), socioeconomic status (CDC. Centers for Disease Control and Prevention, 2023; Kivimäki et al., 2020; Rosengren et al., 2019; Havranek et al., 2015; Kivimäki et al., 2018) – including food insecurity (Kopparapu et al., 2020; Campanera et al., 2023; Ma et al., 2024) –, and physiological and mental stress (Miller and Jones, 2014; Wild, 2012; Mariotti, 2015; Smyth et al., 2018; Ng et al., 2020).

### 2.3 Specific external

Specific external factors include those that have been most extensively studied in epidemiological studies examining exposure and environmental risk factors in disease. These factors include daily exposures attributed to diet, lifestyle factors, medical interventions, occupational and environmental exposures (Wild, 2012). Overall, external factors can be measured using survey instruments and personal sampling (recording). The effect of exposures classified under specific external exposome will be discussed in detail below.

## 3 Specific external exposome and aging healthspan

The most expansive exposure pathway includes ingestion of exogeneous materials ranging from dietary choices, lifestyle factors (i.e., alcohol, tobacco, caffeine), and medical interventions, namely, pharmaceuticals. Many of these exposures are chronic exposures, potentially leading to serious long-term health effects. Another common source of chronic exposures is external exposure to a host of occupational and environmental agents, including radiation, noise, infectious agents (i.e., pathogens), chemical contaminants, environmental pollutants, and additional occupational exposures (i.e., crystalline silica, diesel exhaust, radon, wood dust) (Wild, 2012). While precautions for environmental and occupational exposure limits are in place, these direct or indirect potential occupational and environmental exposures can result in both short- and long-term health effects, leading to health disparities.

Apart from the abovementioned environmental toxicants, some of the other specific external exposures, specifically dietary choices, can have beneficial effects on health and healthy aging. The association between diet and oxidative stress and inflammatory processes has been widely examined. Plant-based diets consisting of anti-inflammatory and antioxidant-rich foods are associated with decreased levels of oxidative stress and inflammatory biomarkers, which have been extensively reviewed elsewhere (Aleksandrova et al., 2021). However, individual components of these diets (vitamins, polyunsaturated fatty acids) and specific metal exposures on healthy aging have not been extensively reviewed and will be discussed below.

### 3.1 Micronutrients

There are many crucial nutrients that are required for endogenous functions that must be obtained throughout the diet. Many of these play crucial roles in supporting various bodily functions, reducing oxidative stress, and maintaining cellular integrity. However, the absorption, metabolism, and utilization of these nutrients can decline with aging, resulting in a loss of protection against cellular processes (Doley, 2017). Aging-related changes may also affect dietary intake in older adults due to increased difficulty chewing and swallowing, changes in taste and smell, poor appetite, and self-restriction, all of which may result in deficiencies in obtaining the recommended dietary allowance of nutrients necessary for physiological functions (Doley, 2017). Conversely, some of these nutrients can trigger aging-related mechanisms such as cell death, oxidative stress, and mitochondrial dysfunction.

#### 3.1.1 Vitamins

Vitamins can be classified as either fat- or water-soluble. Fat-soluble vitamins (A, D, E, K) are stored and accumulated in the liver and lipid tissues. Thus, some fat-soluble vitamins can be toxic if taken in excess. Meanwhile water-soluble vitamins (B and C) are not stored in the body as any excess is excreted and these vitamins need to be consumed regularly to prevent deficiencies (Chernoff, 1995). The Dietary Guidelines for Americans 2020–2025 details the daily nutritional goals for the essential vitamins needed at each age range (Dietary Guidelines for Americans, 2020). However, these guidelines have an upper age of 51+ years and thus do not consider the variability in absorption and utilization that may occur for later aging stages in life. Due to the antioxidant and oxidative stress reducing roles that vitamins play *in vivo*, more comprehensively understanding their effects in aging is of great interest in current geroscience research.

Several vitamins regulate programmed cell death mechanisms, with consequent implications in aging. While the role of vitamins in cell death, cellular senescence, and aging is not the focus of this review, we summarize the findings of vitamins in cell death/cellular senescence and aging in Tables 2, 3, respectively. The main mechanism of vitamin involvement in these processes is throughout their modulation of oxidative stress and interactions with antioxidant enzymes and vitagenes (i.e., genes that maintain cellular homeostasis during stressful conditions). This may potentially impact lipid oxidation, modulating oxylipin synthesis as vitamins A, B, E, and K have been shown to decrease or prevent lipid peroxidation, thereby inhibiting ferroptosis (Shastak et al., 2023; Jakaria et al., 2023; Shan et al., 2021; Moreira et al., 2011; Hu et al., 2021; Chen et al., 2024; Mishima et al., 2022). Interactions with vitamins A and D also result in the upregulation of antioxidant enzymes to prevent cellular senescence (Sirakawin et al., 2024; Sosa-Díaz et al., 2022), while a vitamin K-dependent protein (VKORC1) limits ROS production, also inhibiting cellular senescence (Chen et al., 2024). Likewise, vitamins A, B, E, and K have been shown to directly protect against cognitive decline and age-associated neurodegenerative diseases (Chernoff, 1995; Gruz-Gibelli et al., 2016; Clarke et al., 2007; Vogiatzoglou et al., 2008; Ochi and Takeda, 2015; Mocchegiani et al., 2014; Popa et al., 2021). In summary, vitamins play a critical role in aging and cell senescence, and various levels of exposure could significantly impact human healthspan (Varesi et al., 2022; Diwan and Sharma, 2022).

**TABLE 2:** Interactions between vitamins and cell death/senescence.

Vitamin	Involvement in cell death and cellular senescence	Citations
A	•Vitamin A functions as a chain-breaking antioxidant preventing lipid peroxidation propagation, a peroxyl radical scavenger, a transcriptional regulator of genes involved in oxidative stress pathways, and is capable of inducing autophagy •A Vitamin A metabolite all-trans-retinoic acid (ATRA) has been shown to initiate apoptosis and inhibit ferroptosis	Shastak et al. (2023), Jakaria et al. (2023)
	•Vitamin A can upregulate *skn-1* or *NRF2* (respective *C. elegans* and mammal orthologs) transcriptional levels, inducing downstream antioxidant enzymes such as glutathione S-transferases, leading to a downregulation of proinflammatory and senescence-associated genes	Sirakawin et al. (2024)
B	•Vitamin B6 protects against LPS-induced apoptosis and ferroptosis in the myocardium and alleviates LPS-induced oxidative stress and lipid peroxidation, inhibited the downregulation of antioxidant enzyme expression (Nrf2, NQO1, HO1, GPX4), and reversed the alterations to iron regulatory protein expression (decreased TFR and ferritin, increased FPN1) •Vitamin B12 inhibits high glucose-induced apoptosis in islet β cells, potentially *via* induced autophagy to self-protect cells •Vitamin B12 functions as an efficient scavenger of homocysteine-induced increases in intracellular superoxide, protecting against oxidative stress-related cell death	Shan et al. (2021), Moreira et al. (2011), Zhang et al. (2023a)
	•Vitamin B deficiency leads to increased cellular senescence as Vitamin B6 deficiency increases oxidative stress and systemic inflammation, resulting in accelerated telomere shortening, a hallmark of senescence	Pusceddu et al. (2020)
C	•In a Vitamin C nanoparticle study, millimolar concentrations of this Vitamin C nanoparticles generate H_2_O_2_, disrupting the cellular redox balance, and result in cell death as these nanoparticles were designed to function as a targeted delivery for tumors and cancer cells, to induce cell death in these cells *via* oxidative stress •High doses of Vitamin C induce apoptosis in human gastric cancer cells due to increased intracellular calcium and ROS, and decreased intracellular ATP production in response, leading to mitochondrial dysfunction-induced apoptosis	Chakraborty and Jana (2017), Lim et al. (2016)
	•Low doses of Vitamin C have been shown to protect against cellular senescence by decreasing ROS production, decreasing SA-β-gal activity, indirectly inhibiting p38 MAPK activity, inhibiting p53-induced senescence, inhibiting P13K/AKT cellular senescence pathway, upregulating longevity factors, and recovering cell-cycle arrest by decreasing expression of cell-cycle inhibitors and increasing expression of cell-cycle activators	Kim et al. (1998), Jeong et al. (2017)
D	•Vitamin D has been suggested to promote autophagy by regulating calcium levels, as both an increase or reduction in the homeostatic level of calcium in cells can induce autophagy •The Vitamin D metabolite 1,25-dihydroxyvitain D (1,25(OH)2D) triggers apoptosis in cancer cells and mature adipocytes	Berridge (2017), Sergeev (2014)
	•Vitamin D prevents cellular senescence by upregulating antioxidant enzymes and vitagenes (i.e., genes that maintain cellular homeostasis during stressful conditions) such as Nrf2, Klotho, SIRT, and p53 •Vitamin D promotes a conserved energy profile in cells that can disrupt the high energy required for the senescence inflammatory phenotype •Vitamin D inhibits cellular senescence by interfering at numerous stages of senescence such as inhibiting triggers of oncogene-induced and mitochondrial dysfunction-induced senescence, preventing genomic instability through retrotransposon inhibition, hindering DDR triggers such as telomere attrition and DNA genotoxic stress, and causing cell cycle arrest at the G1 phase •Vitamin D inhibits senescent profile markers throughout the early, classical, and late stages of cellular senescence •In dermal fibroblasts it has been demonstrated that Vitamin D supplementation can decrease senescence-associated mediators (CCL2, IL-6, IL-8) and block p38 MAPK	Sosa-Díaz et al. (2022), Sayegh et al. (2024)
E	•Vitamin E supplementation inhibits ferroptosis in glutathione peroxidase (GPX4)-deficient stem cells, suggesting that Vitamin E functions cooperatively with GPX4 *in vivo* •Some Vitamin E structure-dependent metabolites have been demonstrated to induce apoptosis in various cell lines	Hu et al. (2021), Birringer et al. (2003)
	•Vitamin E protects against senescence by decreasing telomere shortening and decreasing SA-β-gal positive cells •In two cell-based models Vitamin E supplementation could delay, but not arrest, the onset of senescence. Vitamin E may reduce senescence progression by altering cellular proliferation potentially through the p21 pathway	Corina et al. (2019), La Fata et al. (2015)
K	•Vitamin K induces cell death in cancer models *via* apoptosis, autophagy, cell cycle arrest, and ferroptosis in select instances •Vitamin K can be converted to its reduced form (Vitamin K hydroquinone, KH_2_) by ferroptosis inhibitory protein 1 (FSP1), which prevents lipid peroxidation, reducing the amount of NAD(P)H available, thereby inhibiting ferroptosis •Vitamin K epoxide reductase complex subunit 1-like 1 (VKORC1L1) inhibits ferroptosis	Chen et al. (2024), Mishima et al. (2022)
	•Vitamin K inhibits the activation of LOX-12, block activation of NF-κB to limit the production of pro-inflammatory cytokines, and VKORC1 limits ROS production and increases intracellular reduced Vitamin K (KH_2_). KH_2_ then absorbs ROS to protect the phospholipid membrane from oxidation	Chen et al. (2024)

**TABLE 3:** Interactions between vitamins and aging.

Vitamin	Involvement in aging	Citations
A	•Greater storage pools of Vitamin A, decrease in the clearance of Vitamin A retinyl esters, and increased plasma levels of Vitamin A have been observed with aging •A Vitamin A metabolite all-trans-retinoic acid (ATRA) diminishes the production, oligomerization, and deposition of Aβ peptides in AD •ATRA is involved in immune homeostasis and can active pathogenic T cells in inflammatory conditions or autoimmune diseases	Chernoff (1995), Gruz-Gibelli et al. (2016), Raverdeau et al. (2014)
B	•Vitamin B6 deficiency is rare, but has been observed in elderly populations, with one study finding that 10%–45% of the population is deficient in Vitamin B6 •Research suggests that the recommended daily levels of Vitamin B6 for elderly adults should be increased as a depletion-repletion study found that elderly adults (60+ years) needed multiple Vitamin B6 treatments to get back to recommended levels after a deficiency •Vitamin B6 deficiency in elderly populations impacts immune function, with studies demonstrating a decrease in lymphocyte proliferation and IL-2 in Vitamin B6-deficient elderly patients •Absorption of Vitamin B12 can decrease due to age-related decreases in gastric acid secretion, impairing the ability to digest protein-bound Vitamin B12 •Vitamin B12 deficiency is of concern in elderly populations as it has been associated with more rapid cognitive decline, loss of brain volume, dementia, and neuropsychiatric disorders	Chernoff (1995), Clarke et al. (2007), Vogiatzoglou et al. (2008), Ribaya-Mercado et al. (1991), Meydani et al. (1991), Lamers (2022)
C	•Vitamin C is the most potent antioxidant in the body, and it may be involved in protecting against stress-related and degenerative diseases, which particularly affect elderly populations •A significant Vitamin C deficiency has been observed in elderly individuals, especially in those with impaired mobility leading to more sedentary conditions	Chernoff (1995)
D	•In the aging process, Vitamin D may aid against age-related frailty phenotypes by preventing osteoporosis and fractures, supporting muscle function, and aiding in immune health •Vitamin D deficiencies are commonly observed in aging populations as older adults have lower levels of 1,25-dihydroxyvitain D (1,25(OH)_2_D) due to factors such as lower dietary intake, decreased sunlight exposure, and lower production of 25-hydroxyvitamin D (25(OH)D) in response to UVB exposure •Age-related decreases in 7-dehydrocholesterol production in the skin and conversion of 25(OH)D to 1,25(OH)_2_D in the kidneys have also been observed •The expected age-related decrease in renal function may affect Vitamin D conversion and clearance, although aging is not suspected to affect VitD absorption efficiency •Vitamin D regulates many of the processes that drive aging (i.e., autophagy, mitochondrial dysfunction, inflammation, oxidative stress, epigenetics, DNA disorders, calcium and ROS signaling alterations) and thus has been hypothesized to regulate the rate of aging •Under conditions of Vitamin D deficiency, the activity of these aging processes increases, accelerating the rate of aging which can increase the risk for age-related diseases	Chernoff (1995), Berridge (2017), Schoenmakers and Jones (2024), Bikle (2014)
E	•Vitamin E compounds are potent antioxidants, well known for their chain-breaking antioxidant properties that can arrest the lipid peroxidation propagation cycle. Thus, Vitamin E has been suggested to be protective against cognitive decline, maintain skin health, support immune function, and reduce the risk of chronic diseases by combating oxidative stress •The effect of VitE in age-related diseases remains controversial as mammalian models, epidemiological studies, and clinical studies for Vitamin E involvement have demonstrated varying results •In some CVD studies, Vitamin E was either beneficial or had no effect on the incidence of cardiac events. However, some studies concluded that Vitamin E supplementation increased the risk of cardiac events and concluded that high doses of VitE should be avoided •The majority of studies examining the role of Vitamin E in patients with diabetes to prevent CVD also found either no effect or adverse heart effects with supplementation •Vitamin E was found to be beneficial for bone health in rats, but epidemiological studies found no correlation or negative effects of Vitamin E on bone density, and supplementation of the Vitamin E form α-tocopherol may suppress other forms of Vitamin E in the body leading to negative effects on bone formation •Vitamin E has also shown inconsistent results in cancer incidence and development, with one human study finding that Vitamin E supplementation significantly increased prostate cancer risk in healthy men •In neurodegenerative diseases, Vitamin E was especially beneficial in the treatment of AD, with cell and murine models showing a rescuing effect with VitE supplementation. However, clinical results are controversial with most beneficial effects at low doses only in AD	Doley (2017), Ochi and Takeda (2015), Mocchegiani et al. (2014), Zaffarin et al. (2020), Mustacich et al. (2007)
K	•Vitamin K can mitigate some of the common age-associated diseases by ◦ supporting bone health through carboxylation of osteocalcin, which regulates bone formation and mineralization, specifically transporting and depositing calcium in the bones ◦ activating Gla prot1ein to prevent vascular calcification, a factor in CVD ◦ improving insulin sensitivity and acting as an anti-inflammatory through its regulation of osteocalcin in metabolic disorders ◦ acting against oxidative stress- and Aβ-induced apoptosis through carboxylation of the Vitamin K-dependent protein Gas6 to prevent neurodegenerative diseases •Vitamin K deficiencies have been implicated in multiple age-related diseases such as cardiovascular, bone (osteoporosis, osteoarthritis, rheumatoid arthritis), metabolic (diabetes, obesity), neurodegenerative (AD, PD, multiple sclerosis), and pulmonary diseases as well as cancer	Popa et al. (2021), Simes et al. (2020)

The antioxidant capabilities of vitamins have been investigated for their ability to also modulate the lipid profile; thus, influencing aging and age-associated disease risk through this mechanism as well. One study examining variations to lipid classes after multi-micronutrient supplementation (Vitamin A, B1, B2, B3, B5, B6, B7, B9, B12, C, D3, E, calcium, phosphorous, iron, magnesium, and zinc) in children and adolescents found significant reductions in sterol esters (SEs), phosphatidylinositols (PIs), phosphatidylcholines (PCs), and lysophosphatidylcholines (LPCs), and that linoleic, oleic, palmitic, arachidonic, and stearic acid in various lipid classes (SE, PI, PC, LPC, and triacylglycerols) were reduced with supplementation (Chakrabarti et al., 2020). Another study examining long-term antioxidant micronutrient supplementation (Vitamin A, C, E, selenium, and zinc) after 7.5 years identified lower total cancer incidence and all-cause mortality in men but not in women. The authors hypothesize that this sex-based difference may be due to lower baseline antioxidant levels in men (Hercberg et al., 2004). While the interaction between dietary vitamin supplementation and disease prevention has been thoroughly reviewed elsewhere (Rautiainen et al., 2016), current debates state that the benefits of micronutrient supplementation may be lacking among already well-nourished individuals. Two studies, one examining type 2 diabetes and the other examining cognition, also found insignificant benefits with vitamin supplementation (Farvid et al., 2004; Grodstein et al., 2013). Although numerous studies have examined vitamins and aging, the complete involvement of vitamins in maintaining or perturbing healthy aging mechanisms is not entirely understood.

### 3.2 Metals

Humans also require various minerals and trace elements to sustain vital life processes. The “metallome” describes the entirety of metal and metalloid species (elemental distribution, equilibrium concentrations of free metal ions, and free metal content) in a biological system (cellular compartment, cell, tissue type, or organism) (Szpunar, 2004; Williams, 2001; Lobinski et al., 2010). Metals ions such as calcium, chloride, chromium, cobalt, copper, fluorine, iodine, iron, magnesium, manganese, molybdenum, phosphorus, potassium, selenium, sodium, zinc, and more are necessary for physiological processes. It is estimated that one-third of all proteins require a metal cofactor, and therefore the body also contains mechanisms for regulating these metals from catalyzing cytotoxic reactions (Tainer et al., 1991; Finney and O’Halloran, 2003). Many analytical techniques are used to examine the metallome (Szpunar, 2004) due to both their vital roles (regulatory cofactors, electrolytes, structural function, hormone synthesis, antioxidant activity, cell signaling, energy production, and implication in the prevention of several diseases) and negative consequences (highly toxic, involved in pathophysiology of disease and disease risk) (Doley, 2017; Lobinski et al., 2010). Apart from essential metals needed to sustain physiological processes, humans are exposed to a wide variety of other metals in either environmental or occupational settings, which can have serious health outcomes.

#### 3.2.1 Copper (Cu)

Copper is required by the body for a variety of functions and is required for structural and catalytic properties in many enzymes involved in growth, development, and maintenance (Gaetke and Chow, 2003). Copper is also important for the utilization of iron, synthesis of connective tissue, formation of red blood cells, energy production, and antioxidation (Doley, 2017). The majority (85%–95%) of copper in the body is bound to the multi-copper binding protein ceruloplasmin, but the remaining free copper can react generating ROS (Brewer, 2010). Homeostatic regulation of copper absorption and excretion helps prevent toxicity and deficiency (Turnlund et al., 1998). Despite this regulation, copper deficiency and toxicity can occur in the liver, kidneys, brain, and endocrine system (Gaetke and Chow, 2003; Uriu-Adams and Keen, 2005). Copper deficiency can cause a range of symptoms likely due to a decrease in the activity of ceruloplasmin and subsequent iron accumulation and oxidative stress (Uriu-Adams and Keen, 2005). Copper deficiency can also affect the activity of other copper-containing enzymes, non-copper-containing enzymes within the oxidant defense system, and other ROS scavengers (Uriu-Adams and Keen, 2005). The effect of copper deficiency in decreasing the ability to deal with oxidative stress was demonstrated by increased oxidative damage in a copper-deficient rat model (Saari et al., 1990). Copper toxicity also results in oxidative damage, but this effect is suggested to be more of a direct effect of copper itself interacting to cause oxidative stress throughout the body and perturbing the endocrine system (Gaetke and Chow, 2003; Uriu-Adams and Keen, 2005). Copper toxicity in the pathogenesis of age-related diseases such as neurodegenerative diseases, diabetes, and atherosclerosis has been examined and reviewed extensively elsewhere (Brewer, 2010; Uriu-Adams and Keen, 2005). However, the effect of copper in age-associated diseases is thought to be due to free copper accelerating the production of toxic radicals. For neurodegenerative diseases, one study showed that low amounts of copper in distilled drinking water (0.12 parts per million) dramatically increased Aβ plaques and decreased cognitive performance in a cholesterol-fed rabbit AD model (Sparks and Schreurs, 2003). Further, the amyloid precursor protein has copper-binding domains allowing it to function as a copper chaperone in the brain. Copper can also interact with Aβ peptides to produce H_2_O_2_, causing oxidation of Aβ, consequently generating protease-resistant soluble and cross-linked Aβ (Atwood et al., 2000). In humans, those in the highest quintile of total copper intake, combined with a high fat diet, showed an markedly accelerated decline in cognition of at a rate of 19 years over a 6-year period (Morris et al., 2006). A copper-induced cell death (cuproptosis) in which copper exposure results in excessive cellular respiration leading to cell death has recently been described (Chen et al., 2023).

#### 3.2.2 Iron (Fe)

Iron is a vital element for many biological processes including the formation and transport of oxygen in hemoglobin and myoglobin, cellular respiration in the mitochondrial electron transport chain, and DNA synthesis by ribonucleotide reductase (Doley, 2017; Galaris et al., 2019). While excess iron is stored within ferritin in cells, excessive systemic iron can induce hepcidin to sequester iron. Meanwhile, iron deficiency can repress hepcidin expression to regulate the physiological concentrations of iron (Galaris et al., 2019). Iron is tightly controlled at the systemic and cellular levels to prevent iron overload and toxicity, which is associated with liver disease, diabetes, osteoporosis, heart disease, atherosclerosis, and neurodegenerative diseases (Galaris et al., 2019; Puntarulo, 2005). Excess labile iron can result in oxidative stress as iron is a major catalyst in the generation of highly reactive free radicals through the Fenton reactions (Galaris et al., 2019). Increased labile iron and subsequent lipid peroxidation due to ROS production are key features of ferroptosis, an iron-dependent cell death mechanism (Dixon et al., 2012). Age-induced increases in iron due to loss of cellular iron homeostasis mechanisms subsequently increase oxidative stress, contributing to aging-related diseases (James et al., 2015). An iron deficiency can also result in systemic effects, driven by an increase in oxidative stress (Esen Ağar et al., 2021).

#### 3.2.3 Arsenic (As)

Arsenic is a toxic metal and is included in multiple toxic substance and carcinogen lists (Rahman et al., 2018). Arsenic has no known beneficial effects *in vivo*. Humans can be exposed to arsenic from contaminated water, plants (especially in rice), animal sources, and air and soil sources (Rahman et al., 2018). Prolonged exposure to arsenic causes irreversible damage to the body in a range of organs and systems, including the skin and nervous, respiratory, cardiovascular, immune, hepatic, renal, urinary, and endocrine systems (Rahman et al., 2018). Arsenic has been linked with skin, lung, bladder, liver, and kidney cancer (Rahman et al., 2018). While the mechanism of arsenic toxicity is not entirely defined, lipid peroxidation induced by oxidative stress may be responsible (Xu et al., 2017). One study found that arsenic exposure can lead to a decrease in plasma HDL and an increase in plasma LDL, especially at high arsenic concentrations (Zhao et al., 2021). Another meta-analysis found that arsenic can cause oxidative injury by reducing the anti-oxidative capacity, resulting in increased levels of oxidants and decreased levels of anti-oxidants (Xu et al., 2017). Arsenic may also contribute to multisystem decline through an induced acceleration of physiological aging processes (Sorrentino et al., 2014). In an evaluation of epidemiological data, arsenic exposure was found to accelerate DNA methylation (a measure of biological aging), consequently affecting the risk of age-related morbidity. Specifically, arsenic exposure increased the risk of CVD incidence, CVD mortality, and all-cause mortality (Jiang et al., 2023). Arsenic also triggers nonalcoholic steatohepatitis (NASH) by inducing ferroptosis via the Mfn2/IRE1α-ACSL4 pathway, contributing to the lipid metabolism disturbances associated with NASH (Wei et al., 2020).

#### 3.2.4 Cobalt (Co)

Cobalt exposure is typically through food intake, mainly through fish and vegetables, or from inhalation of airborne particles from industrial emissions. Cobalt-contaminated water is very rare (Lang et al., 2009). Chronic exposure to cobalt is associated with cardiac abnormalities, gastrointestinal disturbances, lung disease, and cancer (Lang et al., 2009). A study of data from the NHANES found that cobalt exposure, even within normal range, may be a risk factor for age-related mobility effects (Lang et al., 2009). Cobalt exposure is associated with oxidative stress, as *in vivo* and *in vitro* lung samples exposed to cobalt showed an increase in the oxidized form of glutathione (GSSG), decreased reduced form of glutathione (GSH), and increased activity of the pentose phosphate pathway (Lewis et al., 1991). Oxidative stress due to cobalt may also be a mechanism underlying the pathogenesis of PD (Uversky et al., 2002). Cobalt, in the presence of calcium, can also trigger mitochondrial oxidative stress inducing apoptosis (Battaglia et al., 2009).

#### 3.2.5 Cadmium (Cd)

Exposure to the toxic heavy metal cadmium is largely through tobacco smoke, diet, or occupational exposure (Zhang et al., 2023b). Cadmium accumulates in the body and has a biological half-life of 10 years, resulting in chronic endogenous exposure. Therefore, urine cadmium functions as a biomarker of long-term cadmium exposure (García-Esquinas et al., 2015). Cadmium contributes to several age-related diseases such as diabetes, CVD, kidney disease, and bone disease (osteoarthritis, osteoporosis). It also contributes to oxidative stress by stimulating cytokine production, elevating levels of inflammation, and increasing mitochondrial damage (Zhang et al., 2023b; García-Esquinas et al., 2015). An epidemiological study examining NHANES data found that cadmium exposure is associated with increased whole-body phenotypic aging, and smoking (one of the main sources of cadmium exposure) was positively associated with aging (Zhang et al., 2023b). Another study using NHANES data demonstrated that urinary cadmium levels were negatively correlated with telomere length. Telomere shortening is a biomarker of aging and is capable of triggering cellular senescence (Xia et al., 2022). Oxidative stress induced via cadmium may be involved in the mechanisms underlying the development of PD (Uversky et al., 2002). In cancer models, acute cadmium exposure can cause toxic effects. However, chronic exposure can lead to an adaptive reduction in ROS production, at the expense of aberrant gene expression, leading to cadmium-induced carcinogenesis (Liu et al., 2009). Cadmium has also been shown to induce multiple forms of cell death, including apoptosis, necroptosis, autophagy, and pyroptosis (Messner et al., 2015; Zhou et al., 2022).

#### 3.2.6 Selenium (Se)

The trace element selenium is required by the body as it is an element in amino acids and enzymes (selenoproteins) where it contributes to antioxidant defense, immune functions, and metabolic homeostasis (Bjørklund et al., 2022). Selenium is a crucial cofactor for glutathione- and iodine-containing enzymes and is involved in thyroid function (Doley, 2017). However, selenium has a narrow margin of beneficial effects in the body as selenium toxicity can result in pro-oxidant functions and liver, lung, and neurological damage. Selenium deficiency can cause oxidative stress, chronic inflammation, endocrine and immunity dysregulation, reduced life expectancy, acceleration of aging processes, and increased vulnerability to age-related disease (Bjørklund et al., 2022). Selenium deficiency has also been reported to increase in proportion to age and age-related diseases (Savarino et al., 2001). Multiple studies, extensively reviewed elsewhere, examining older adults (largely centenarians) found that those with longer lifespans had either higher serum levels of selenium or much lower percentages of selenium deficiencies, suggesting that inadequate selenium could adversely affect optimal health in aging populations (Bjørklund et al., 2022). Another study found that elderly adults with low levels of selenium experienced significantly higher rates of all-cause mortality than elderly adults with high levels of selenium (Giovannini et al., 2018). It is suggested that the role of selenium in aging may be due to selenoprotein involvement in redox homeostasis and anti-inflammatory activities (Cai et al., 2019). Selenium has also been linked to cellular senescence, as a downregulation in the expression of selenoproteins during selenium deficiency can activate senescence (Legrain et al., 2014) and low levels of selenium can result in shortened telomere length (a hallmark of senescence), which can be rescued by selenium administration (Schoenmakers et al., 2010; Liu et al., 2004). The selenoprotein GPX4 is an essential regulator of ferroptosis and directly prevents uncontrolled phospholipid peroxidation (Ito et al., 2024).

#### 3.2.7 Lead (Pb)

Lead is a well-known toxic element, and exposure to lead can come from a variety of sources such as food, water, paint, air, and occupational settings (Vig and Hu, 2000). Previous lead exposures may alter the aging process as lead can be stored in reservoirs in bones which can protect the body from lead toxicity. However, age-related bone loss can release this stored lead into the bloodstream (Vig and Hu, 2000). A mix of epidemiological, clinical, and autopsy studies demonstrated a trend of increasing lead in the blood or bones with aging, although many results were not statistically significant (Vig and Hu, 2000). Low level lead exposure can result in hypertension, peripheral artery disease, kidney and neurodegenerative diseases, and cognitive impairment (Ahamed and Siddiqui, 2007). Lead may induce oxidative stress in the pathophysiology of these diseases, leading to oxidative damage in the heart, liver, kidney, reproductive organs, and brain (Ahamed and Siddiqui, 2007). A study examining oxidative stress and lead toxicity in lead-exposed workers found that there is a disruption to the oxidant/antioxidant balance in the workers compared to controls (Gurer-Orhan et al., 2004). Additionally, an accumulation of δ-aminolevulinic acid could potentially be a source of ROS in the pathophysiology of lead toxicity (Ahamed and Siddiqui, 2007). Lead binds to the membrane of cells, acting as a stimulant for lipid oxidation which can lead to alterations in the lipid membrane composition. These alterations can affect membrane integrity, permeability, function, and susceptibility to lipid peroxidation (Adonaylo and Oteiza, 1999), suggesting that lipids are highly involved in lead-induced toxicity.

#### 3.2.8 Mercury (Hg)

Mercury is a toxic element that has been used for centuries. Mercury exposure can be due to contact with a variety of sources such as soil, air, water, plants and animals, and can still be found in some older fluorescent lightbulbs, thermometers, and batteries (Kumari et al., 2023). A major issue of mercury is its bioaccumulation and biomagnification properties, as the most hazardous form (methylmercury) is found most frequently due to its quick absorption and slow excretion (Kumari et al., 2023). Mercury poisoning has been associated with many health effects in both male and female reproductive systems, *in utero* development, and deleterious effects on the central nervous, renal, pulmonary, immune, gastrointestinal, and cardiovascular systems (Kumari et al., 2023; Rice et al., 2014). Mercury can cross the blood brain barrier, causing oxidative stress, mitochondrial dysfunction, and neuroinflammation, resulting in a wide range of neurological effects (Kumari et al., 2023; Rice et al., 2014; Teixeira et al., 2018). Oxidative stress due to mercury exposure can also induce cell death by cytotoxicity and apoptosis (Teixeira et al., 2018). Mercury can also predispose tissues to aging and one study found that the percentage of people with inorganic mercury in the organs that commonly contain mercury (brain, kidney, thyroid, anterior pituitary, adrenal medulla and pancreas) increased with aging, with the highest levels of mercury in all six organs peaking in the 61–80 years group. Mercury levels slightly decreased in these organs, apart from adrenal medulla, suggesting that those who “aged successfully” likely had lower lifetime mercury exposure giving them an advantage in aging. However, these six organs have also been implicated in the process of accelerated aging; thus, mercury may accelerate aging processes in these organs via a variety of mechanisms. The presence of mercury in multiple organs may also offer an explanation to why certain disorders often occur together (Pamphlett, 2021).

Overall, exposure to metals over the lifespan and accumulation of heavy metals *in vivo* can result in toxic effects on many of the body’s physiological processes. However, metal exposure, as discussed above, can catalyze cytotoxic reactions and affect oxidative stress, with more severe influential effects on oxidative stress when in combination with aging-related dysregulation of these physiological processes (Szpunar, 2004; Kim Dwon et al., 2022). The consequent oxidative stress resulting from metal exposure can also influence the lipid profile, with previous findings demonstrating that lead, mercury, iron, zinc, and arsenic affect dyslipidemia (measured via cholesterol, low-density lipoprotein cholesterol, and high-density lipoprotein cholesterol) (Kim Dwon et al., 2022; Luo et al., 2022). Meanwhile, other studies have examined changes in the metallome over aging in a variety of tissues (Zhang et al., 2020; Takahashi et al., 2001), with Zhang et al. observing that mice on calorie restriction had shifted but similar trends to aging mice not on calorie restriction, demonstrating that changes in metabolism may slow aging (Zhang et al., 2020). Despite numerous studies examining metals on aging processes, as well as effects of metal deficiency and toxicity on age-related diseases, reviewed elsewhere (Wessels et al., 2015), the extent of metal involvement in oxidative stress, lipid profile modulation, and cell death influencing healthy aging mechanisms are still not entirely understood.

## 4 Oxidative lipid metabolism and aging healthspan

One of the major macronutrients in the human diet is fats, which are broadly defined as lipids. Lipids encompass cholesterol, saturated fatty acids, mono- and polyunsaturated fatty acids, and trans fats, which can be found as free fatty acids or incorporated into triglycerides and phospholipids. Lipids are key biological molecules responsible for forming cellular membranes, providing cellular energy metabolism, and acting as signaling molecules or affecting membrane fluidity in signal transduction (Mutlu et al., 2021). The subsequent sections will discuss the interactions of the exposome on oxylipin metabolism, including PUFAs, CYPs, EH (specifically sEH), LOX, and COX, followed by exploration of the effects of each section of oxylipin metabolism on aging and senescence.

### 4.1 Polyunsaturated fatty acids

Polyunsaturated fatty acids (PUFAs) are composed of a carboxylic acid head group and a long-chain carbon tail containing at least two carbon-carbon double bonds. These molecules are critical in an extensive list of physiological processes as they can modulate membrane channels and proteins, regulate gene expression via nuclear receptors and transcription factors. PUFA derivatives and glycerophospholipids containing PUFAs can also serve as bioactive signaling molecules and as the building blocks of membrane lipids (Sarparast et al., 2020; Mozaffarian and Wu, 2011; Harayama and Shimizu, 2020). PUFAs are found in all cell membranes and tissues in the body, and are particularly known for their roles in the health, function, and protection of the cardiovascular system, brain, and skin barrier as well as in insulin sensitivity (Mititelu et al., 2025). Specifically, docosahexaenoic acid (DHA), an ω-3 PUFA, is an essential membrane component in the brain, retina, and nervous system, comprising around 40%–60% of the membrane fatty acids (Querques et al., 2011; Dyall, 2015). ω-3 PUFA supplements are suggested to be beneficial for cognitive function, vision, and neurological health. Eicosapentaenoic acid (EPA) supports cardiovascular health and global inflammatory responses through its roles in reducing triglyceride levels, improving endothelial function, and preventing excessive platelet aggregation. Meanwhile, γ-linolenic acid (GLA), an ω-6 PUFA, plays a minor role in the body’s anti-inflammatory processes, and is vital in supporting skin barrier structure and function. Dihomo- γ-linolenic acid (DGLA) functions as a precursor to various anti-inflammatory molecules, while arachidonic acid (AA) and its downstream metabolites are essential in cellular signaling, immune response, and tissue repair (Mititelu et al., 2025). Overall, ω-3 PUFA metabolites tend to be regarded as pro-resolving mediators, while ω-6 PUFA metabolites are generally considered to be pro-inflammatory. However, this consensus is not universally true, as some ω-3 epoxy-PUFA-metabolites promote allergic responses by attenuating mast cell activation while an ω-6 PUFA-metabolite (lipoxin A_4_) has been shown to be more pro-resolving (Dalli and Serhan, 2019; Shimanaka et al., 2017). The role of PUFAs in the pathology of multiple age-related diseases has been investigated due to their ubiquitous presence, multiple metabolism pathways, and potency despite their low abundance *in vivo*.

#### 4.1.1 Biosynthesis pathway and dietary intake of PUFAs

**FIGURE 2:**
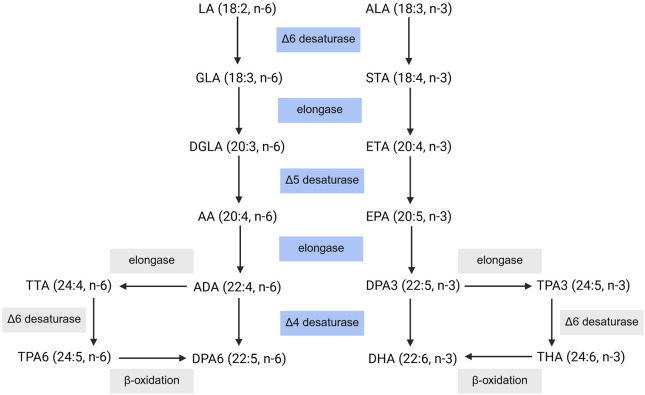
PUFA Biosynthesis Pathway. Dietary intake of LA and ALA can be converted into their respective downstream PUFAs. LA: linoleic acid, GLA: γ-linolenic acid, DGLA: dihomo- γ-linolenic acid, AA: arachidonic acid, ADA: adrenic acid, DPA6: docosapentaenoic acid, TTA: tetracosatetraenoic acid, TPA6, tetracosapentenoic acid (ω-6), ALA: α-linolenic acid, STA: stearidonic acid, ETA: eicosatrienoic acid, EPA: eicosapentaenoic acid, DPA3: docosapentaenoic acid, DHA: docosahexaenoic acid, TPA3: tetracosapentenoic acid (ω-3), THA: tetracosahexaenoic acid. Created with BioRender.com.

**FIGURE 3:**
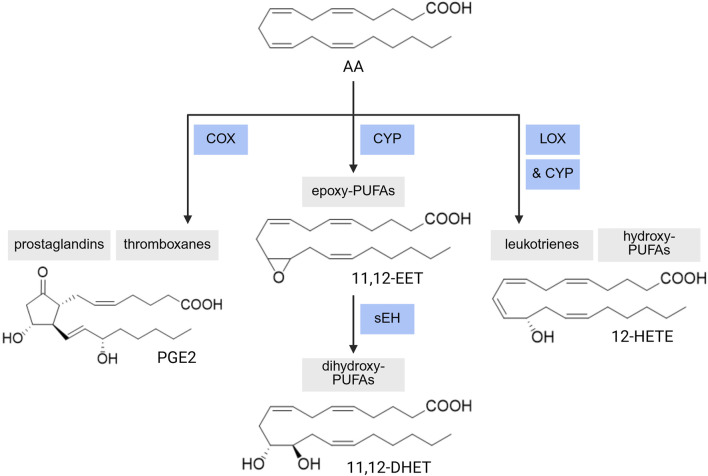
PUFA Enzymatic Metabolism. A range of PUFA metabolites can be produced via CYP-sEH (cytochrome P450-soluble epoxide hydrolase), COX (cyclooxygenase), or LOX (lipoxygenase)-mediated reactions. AA: arachidonic acid, EET: epoxyeicosatrienoic acid, DHET: dihydroxyeicosatrienoic acid, HETE: hydroxyeicosatetraenoic acid, PGE2: prostaglandin E2. Created with BioRender.com.

Mammals are capable of endogenously synthesizing the respective downstream ω-3 and ω-6 PUFAs from linoleic acid (LA) and α-linolenic acid (ALA); however, LA and ALA must be obtained from the diet (Figure 2). Through elongase and desaturase enzymes, these PUFAs can be produced and thus available for metabolism by cytochrome P450 enzymes, lipoxygenase, and cyclooxygenase (Figure 3), into various oxylipins which will be discussed later in this review. The PUFA biosynthesis pathway is limited. One study found that the conversion of LA to DGLA was between 1.5%–2.1% while the conversion of LA to AA was between 0.3%–0.6% (Demmelmair et al., 1999). Likewise, the conversion of ALA to EPA and DHA was found to be ∼8% and 0%–4% in young men and 21% and 9% in young women, respectively (Burdge et al., 2002; Burdge and Wootton, 2002). Thus, mammals are heavily dependent on the dietary intake of PUFAs. However, the conversion of PUFAs is variable between populations (Mathias et al., 2011). Previous studies found higher levels of circulating long chain-PUFAs (LC-PUFAs) in African Americans than European Americans. As well, African Americans have higher levels of AA over DGLA while European Americans have higher levels of ALA over EPA and ω-3 docosapentaenoic acid (DPA3). Likewise, African American populations were shown to have higher levels of AA and increased AA/DGLA and AA/LA ratios relative to Hispanic American populations. These differences can be partially explained by variations in the frequencies of *FADS* (fatty acid desaturase) genetic variants between different ancestry populations, with haplotype D (found in African populations) being associated with increased biosynthesis of LC-PUFAs (Mathias et al., 2011; Harris et al., 2019; Lemaitre et al., 2011; Ameur et al., 2012; Yang et al., 2023). Genetic variants in *FADS1* and *EVOVL2* in a Tunisian population were found to increase levels of AA and the AA/DGLA ratio, increasing the risk for AD in individuals with these genetic variants (Hammouda et al., 2020). Likewise, a recently published review detailed how different genetic variants within the *FADS1*, *FADS2*, and *EVOVL2* genes can influence atherosclerotic cardiovascular disease risk differently based on the variant (Liu et al., 2024).

#### 4.1.2 Evidence of the effects of the exposome on PUFAs

External exposomal exposures can directly and indirectly affect the microbiome, leading to dysbiosis, which can affect the functions of the microbiome. The net result is a downstream effect on the internal exposome, which influences human health and disease (Merra et al., 2024; Losol et al., 2023). The effect of the exposome on the microbiome can influence the absorption and biotransformation of PUFAs by gut microbiota, modulating lipid signaling and composition in the body. Some studies also examined the interplay of the exposome and PUFAs, with most studies focusing on the interaction of downstream metabolizing enzymes and their products after external exposures. However, one study examining asthma outcomes with dietary PUFAs showed that higher ω-6 PUFA intake exacerbated asthma severity in response to indoor particulate matter (PM ≤ 2.5) while higher ω-3 PUFA intake alleviated asthma symptoms due to PM (Brigham et al., 2019). Another study found that habitual dietary intake of ω-3 PUFAs has a protective effect against air pollution-induced (PM ≤ 2.5, ozone) cardiovascular effects (Chen et al., 2022). Reviewed elsewhere, increased dietary intake of ω-6 can promote hepatotoxic responses to persistent organic pollutants (i.e., polychlorinated biphenyl (PCB)) including oxidative stress. Meanwhile, changing the ratio of ω-6/ω-3 or increasing ω-3 intake can protect against the PCB-induced inflammation (Hennig et al., 2012).

#### 4.1.3 Evidence of the effects of PUFAs on aging and senescence

Characterizing an association between the dysregulation of lipid metabolism and aging has been a recent focus within the field. A study examining fatty acids in the blood of adults (18–64), older adults (65–89), and long-lived individuals (LLIs, 90–111) found that LLIs and older adults had a reduction in the ω-3 index compared to adults. Interestingly, EPA and DHA levels were similar between adults and LLIs but not older adults, while DPA3 showed a significant decrease with age. However, the authors also note that aging may not modulate ω-3 PUFAs, but instead an increase in ω-3 PUFAs levels may promote longevity. Conversely, for ω-6 PUFAs, higher levels of DGLA were observed in LLIs compared to adults and older adults while LA and AA decreased with age (Ali et al., 2022). An additional study examined the relationship between PUFAs and markers of oxidative stress and inflammation with aging. Blood ω-3 PUFAs and ω-3 index values were associated with increased C-reactive protein levels in male LLIs. ω-3 PUFAs were positively associated with Trolox equivalent antioxidant capacity (TEAC) in male LLIs, while the AA/EPA ratio was negatively associated with TEAC values in female LLIs. Increased levels of total ω-3 PUFAs and ω-3 index were also associated with increased MDA concentration in male LLIs, while total ω-6 PUFAs had a significant inverse correlation in female LLIs (Aiello et al., 2024). Furthermore, genetic knockout of Elovl2 (elongase of very long chain fatty acids 2) in mice accelerated aging phenotypes and aging hallmarks, suggesting a role for Elovl2 in aging. Reduction or loss of Elovl2 also results in an increase in fatty acid synthesis, leading to changes in mitochondrial energy metabolism and accumulation of fatty acid precursors for PUFA synthesis in the ER causing ER stress (Li et al., 2022). Other studies in *Caenorhabditis elegans* demonstrated that changes in dietary PUFAs and oxidative state can modulate the lifespan (Watts, 2016). Understanding the lipid signaling involvement of all fatty acids in aging is also of great interest in the field and has been reviewed elsewhere (Mutlu et al., 2021). Additionally, changes in lipid composition for the various PUFA forms (i.e., triglycerides, phospholipids, etc.) during cellular senescence have been reviewed elsewhere (Hamsanathan and Gurkar, 2022).

### 4.2 Cytochrome P450s

Cytochrome P450 monooxygenases (CYPs) constitute an extensive family of heme-thiol containing monooxygenases that are responsible for metabolizing xenobiotics such as drugs, pesticides, carcinogens, etc. (Degtyarenko and Archakov, 1993; Bernhardt, 2006; Werck-Reichhart and Feyereisen, 2000). CYPs are also involved in the metabolism of many physiologically important endogenous compounds including steroid hormones, cholesterol, vitamins, and fatty acids (Bernhardt, 2006). Within the body, CYPs are mainly concentrated within the liver due to their role in xenobiotic and endogenous metabolism but are ubiquitously expressed in most tissues including the kidneys, intestines, brain, heart, lung, and skin (Anzenbacher and Anzenbacherová, 2001; Preissner et al., 2013). Within cells, CYPs are predominately membrane-bound on the ER or mitochondria, with the ability to move along the membrane surface as the catalytic domain remains on the cytosolic side but is anchored by the N-terminal transmembrane on the luminal side (Šrejber et al., 2018).

#### 4.2.1 Range of CYP families

Within humans, 57 genes expressing various CYP enzymes have been identified, which can be clustered into 18 families with 43 sub-families (Nelson, 2009). The 57 CYP genes identified from the human genome project gave new insight into the extent of CYP isoforms, with many of these enzymes covering a large range of functions, although the role of some isoforms remains unknown (Ingelmansundberg, 2005). In this review, we will only focus on the CYP isoforms related to PUFA metabolism. Many CYP isoforms have been extensively studied for their role in PUFA metabolism, as CYPs can exert both epoxidation (forming epoxy-PUFAs) and hydroxylation (forming hydroxy-PUFAs) on PUFAs as substrates. The extent of preference for PUFA substrates and the type of reaction have been extensively covered elsewhere (Sarparast et al., 2020).

Overall, CYP2B, 2C (2C8, 9, 18, 19, 23), and 2J (2J2, 3, 5, 9) are the subfamilies that predominately generate epoxy-PUFAs (Sarparast et al., 2020). On the other hand, CYP1A (1A1, 2), 4A (4A1, 11, 12A), and 4F (4F2, 3A, 3B, 8, 12) subfamilies produce mostly hydroxy-PUFAs (Sarparast et al., 2020). However, many of these subfamilies will produce the epoxy- or hydroxy-PUFAs as minor products, respectively. There are some isoforms outside of these subfamilies which are also involved in PUFA oxidative metabolism such as CYP3A4 (mainly epoxidation), CYP2E1 (both reactions), and 2U1 (mainly hydroxylation) (Sarparast et al., 2020). The catalytic cycle of CYP reactions (Denisov et al., 2005; Guengerich, 2018; Meunier et al., 2004) is generally accepted for most P450-catalyzed oxidations; however, specific steps and their respective intermediates within the catalytic cycle have proven difficult to characterize. There are some variations to the cycle for the distribution of charge on the iron atom, the iron-oxo bond, and the porphyrin ring (Guengerich, 2018).

#### 4.2.2 Evidence of the effects of the exposome on CYP

Environmental pollutants have been shown to affect the expression of CYP isoforms. 2,3,7,8-tetrachlorodibenzo-*p*-dioxin (TCDD) was found to induce CYP1A1 gene expression and enzyme activity. Polycyclic aromatic hydrocarbons (PAHs) can induce the expression of CYP1A1, CYP1A2, and CYP1B1 (Ibrahim et al., 2019; Rao and Kumar, 2015). Long-term exposure to environmental factors can lead to methylation, acetylation or histone modification, affecting CYP gene expression (Jin et al., 2023). Internal exposomal factors, especially stress, can also significantly affect the expression of CYP isoforms. In animal stress models, stress could downregulate the expression of CYP2E1 and CYP2B, while upregulating the expression of CYP1A, CYP2A, CYP2C, CYP2D, and CYP3A isoforms (Konstandi et al., 2014). In rats, some isoforms such as CYP1A2, CYP2B1, and CYP2E1 are expressed at higher levels in young rats while CYP1A1 is only expressed at early stages of development. Similarly, the expression of CYP2C11 and CYP3A2 drastically increased during puberty in rats then declined again with aging (Yun et al., 2010). The stress- and age-related effects on CYP expression could be due to negative effects on and decline in the activity of hormonal systems that are involved in CYP regulation (Konstandi and Johnson, 2023). Human studies have not largely demonstrated significant changes in CYP isoform expression patterns. However, some studies found slight decreases in CYP isoform activity with aging (Konstandi and Johnson, 2023).

Changes to CYP isoform expression result in imbalances in epoxy- and hydroxy-PUFA levels, causing systemic effects due to the range of these metabolites’ activity and contributing to inflammaging. Epoxy-PUFAs are generally considered more anti-inflammatory, while hydroxy-PUFAs are more pro-inflammatory and have been implicated in multiple diseases (Hwang et al., 2017). Environmental exposures that generate ROS can also non-enzymatically react with PUFAs to form hydroxy-PUFAs, further disrupting homeostasis (Zhu et al., 2021).

#### 4.2.3 Evidence of the effects of CYP on aging and senescence

CYP metabolism of unsaturated fatty acids produces bioactive lipid mediators, which impact aging and senescence. Numerous studies have examined CYP-metabolites in age-associated NDs. One study reported increased levels of LA-derived hydroxy-PUFAs (HODEs) in the plasma and erythrocytes of patients with AD, which are positively correlated with clinical severity (Yoshida et al., 2009). Another study examining lipid mediators in plasma and cerebrospinal fluid between AD and healthy individuals, including analysis with strong predictive and discriminant models, identified differences in CYP/sEH and acylethanolamide metabolism that could serve as predictors of AD. Specifically for CYP/sEH metabolism, the partial least squares discriminant analysis performed on the samples found higher levels of 9,10-EpOME and 12,13-EpOME in the cerebrospinal fluid and a decrease in the ratio of 12,13-DiHOME/EpOME in the plasma in the AD group compared to the control group (Borkowski et al., 2021). In *postmortem* cerebral cortex samples, a positive correlation between various CYP-derived oxylipins and clinical phenotypes of AD were found, including 5-, 9-, and 12-HETE, 12-HEPE, 13-HpODE, 5,6- and 11,12-DHET, and 14,15-DiHETE (Kurano et al., 2022). Meanwhile, levels of oxidized LA metabolites were greatly increased in advanced lesions in *postmortem* aortas of patients with atherosclerosis (Harland et al., 1971).

CYP-mediated reactions can also produce ROS, leading to oxidative stress. Increased ROS production due to age-related dysregulation of CYPs contributes to DNA damage, protein oxidation, and lipid peroxidation all of which accelerate aging and trigger cellular senescence (Chaudhary et al., 2009). CYP isoforms found within the mitochondria also exacerbates local oxidative stress, both impairing mitochondrial function (a hallmark of aging) and promoting cellular senescence (Hamsanathan and Gurkar, 2022; Chaudhary et al., 2009). The involvement of CYP metabolites in cellular senescence is relatively understudied. However, epoxy-PUFAs generated from CYP enzymes have shown effects similar to the beneficial roles of senescent cells, such as involvement in wound healing, tissue repair, and tumor suppression and reduced proinflammatory factor production (Hamsanathan and Gurkar, 2022). CYP-derived epoxy-PUFAs protect against mitochondrial dysfunction resulting in cellular senescence and suppress pro-inflammatory SASP in cardiac models (Yousef et al., 2024).

One study examining changes between age and CYP levels and activity in the liver found that CYP activity decreased after the age of 40 (Sotaniemi et al., 1997). Serum antipyrine, which is metabolized by at least 10 CYP isoforms, is used to measure *in vivo* hepatic CYP activity. Clearance of antipyrine declined linearly after age 40, likely due to age-related changes in CYP expression or activity. Meanwhile the half-life of antipyrine increased linearly after age 30, likely due to age-related changes in liver drug distribution and elimination processes (Sotaniemi et al., 1997). Changes in liver CYP metabolizing activity with aging can result in changes to hormone levels, influencing additional age-related changes. In men, testosterone levels are highest in young age and decline throughout the lifespan, while reduced CYP activity in women may lower estrogen levels in postmenopausal women, impacting bone density, cardiovascular, and neurological health (Sotaniemi et al., 1997; Tsuchiya et al., 2005). Age-related increases or decreases of specific CYP isoforms and their activity can result in dysregulated steroid hormone synthesis and metabolism, leading to excessive accumulation or deletion of various hormones as CYP isoforms within the CYP11, CYP17, CYP19, and CYP21 families are involved in steroid biosynthesis. This dysregulation can also result in ROS production, where the ROS can then promote cell death or cellular senescence. The dysregulation of CYP metabolism in endocrine signaling and its impact on human disease has been more deeply reviewed elsewhere by Hossam Abdelmonem et al. (Hossam et al., 2024).

### 4.3 Epoxide hydrolase(s)

Epoxide hydrolases (EHs) are vastly important in both detoxification, catabolism, and regulatory signaling pathways within the human body (Gautheron and Jéru, 2020; Morisseau and Hammock, 2005). These enzymes metabolize a range of endogenous and exogenous compounds containing an epoxide motif, including pharmaceuticals, toxins, hormones, and epoxy-PUFAs, to their corresponding 1,2-diols (Morisseau and Hammock, 2005; Harris et al., 2008). Within mammals, seven EHs have been identified: microsomal epoxide hydrolase (mEH, encoded by *EPHX1*), soluble epoxide hydrolase (sEH, *EPHX2*), epoxide hydrolase 3 (EH3, *EPHX3*), epoxide hydrolase 4 (EH4, *EPHX4*), hepoxilin hydrolase, leukotriene A4 hydrolase (*LTA4H*), and cholesterol epoxide hydrolase (Sarparast et al., 2020; Gautheron and Jéru, 2020). To date, no corresponding genes have been identified for hepoxilin hydrolase and cholesterol epoxide hydrolase. However, an α/β-fold hydrolase EH subfamily categorizes mEH, sEH, EH3, and EH4 together from the remaining three EHs (which can be categorized separately based on their catalytic mechanism and substrate preferences) (Decker et al., 2009).

#### 4.3.1 sEH

Overall, mEH and sEH have received significantly more attention than EPHX3/4 and thus, their functions are better understood. mEH, EPHX3, and EPHX4 are out of the scope of this review and have been discussed elsewhere (Sarparast et al., 2020; Decker et al., 2009; Decker et al., 2012). Soluble epoxide hydrolase (sEH, *EPHX2*) can be found in the cytosol and peroxisomes of cells. sEH is a bifunctional enzyme with an N-terminal region responsible for phosphatase activity and a C-terminal region responsible for epoxide hydrolase activity, connected by a proline-rich linker (Harris and Hammock, 2013). sEH is ubiquitously expressed and found at its highest concentrations in the liver and kidney, and sEH regulates important physiological processes in the brain, lungs, and heart (Harris and Hammock, 2013). sEH has specifically been shown to be the main enzyme that degrades epoxy-PUFAs (Bylund et al., 1998), especially at high rates of epoxide formation (Edin et al., 2018). The remaining three EH isoforms are also involved in metabolizing epoxy-fatty acids but at a lower rate. sEH hydrolyzes epoxy-fatty acids and generates trans 1,2-diols with no enantiomeric selectivity. On the other hand, mEH is less active against epoxy-fatty acids (Zeldin et al., 1995), but mEH hydrolyzes these substrates with high enantiomeric selectivity (Zeldin et al., 1993; Summerer et al., 2002).

The mechanism of this catalysis is performed in three steps, along with a contribution from four amino acid residues (Sarparast et al., 2020; Gautheron and Jéru, 2020; Morisseau and Hammock, 2005). Within the substrate binding pocket of sEH, the epoxide substrate is bound through hydrogen bonds with the two tyrosine residues, and the binding of the substrate is further enhanced by the hydrophobic interaction with the protein binding pocket. The epoxide is then activated by the hydrogen bond with the tyrosine residues and opened by a nucleophilic attack of the aspartate (which is orientated and activated by a histidine residue, a second carboxylic acid amino acid, and potentially other amino acids located in the catalytic center) on one of the epoxide carbons forming an enzyme-substrate ester intermediate. Following this, the histidine residue relocates away from the ester, in which a water molecule is then activated by the acid-histidine pair. This activated water then hydrolyzes the ester intermediate resulting in the diol product and original enzyme. The mechanism of mEH conversion of epoxides to diol is highly similar, with differences only including a change in the orientating amino acid and the position of the amino acid in both proteins’ sequence (Morisseau and Hammock, 2005). EH3 and EH4 follow the same mechanism and use the same orientating amino acid as mEH, but likewise have differences in the position of the amino acids involved (Morisseau and Hammock, 2005).

#### 4.3.2 Evidence of the effects of the exposome on sEH

Various environmental factors such as cigarette smoke, PAHs (3-methylcholanthrene, benzo(a)pyrene), and pharmaceuticals (ethinyl estradiol, tamoxifen, raloxifene, isoproterenol) have been examined for effects on sEH expression, with many of these resulting in an upregulation of sEH, shifting the balance toward increased dihydroxy-PUFAs to epoxy-PUFAs ratio (Harris and Hammock, 2013). Exposures to LPS, streptozotocin, and monocrotaline also resulted in downregulation of sEH, which reduces the conversion of epoxy-PUFAs to dihydroxy-PUFAs, preserving the anti-inflammatory properties of epoxy-PUFAs (Harris and Hammock, 2013). External and internal factors that induce oxidative stress can also activate sEH via oxidative intra-disulfide formation. This specific activation not only results in increased specific activity of sEH, but also increases the substrate affinity and catalytic efficiency of sEH (Charles et al., 2021).

#### 4.3.3 Evidence of the effects of sEH on aging and senescence

The precursor metabolites of sEH (epoxy-PUFAs) mediate vasodilation, reduce inflammation, attenuate oxidative stress, and block endoplasmic reticulum stress response. Thus, the conversion of epoxy-to dihydroxy-PUFAs can diminish the beneficial effects of epoxy-PUFAs (Griñán-Ferré et al., 2020). Inhibiting sEH has shown beneficial effects in a variety of age-related diseases such as diabetes (Lee et al., 2020; Anita and Swardfager, 2022), atherosclerosis (Piper and Garelnabi, 2020; Kim SA. et al., 2022), and neurodegenerative diseases (Griñán-Ferré et al., 2020; Ren et al., 2018). sEH directly contributes to aging processes in the colon by enhancing ER stress and cellular senescence (Wang et al., 2023). Finally, genetic knockout of sEH demonstrated beneficial effects following cerebral ischemia in reproductively senescent female mice (Zuloaga et al., 2015).

### 4.4 Lipoxygenase

Lipoxygenases (LOXs) constitute a family of non-heme iron-containing dioxygenases that catalyze the stereoselective monooxygenation of PUFAs, with their nomenclature typically denoting their positional specificity of oxygen insertion (Kuhn and Thiele, 1999; Czapski et al., 2016). There are five LOXs found in mammals: 5-, 8-, 12-, 15-LOX and LOX-3 in which LOX-3 does not follow the oxygen-insertion trend or nomenclature. While the structures of the various LOX isoforms are similar to each other, differences in substrate cavity, coordination of histidine residues or variations to the non-heme iron in the catalytic center can affect their substrate specificity (Mashima and Okuyama, 2015). Very little is known about the location of most LOX isoforms. However, current understanding suggests that some isoforms can be found in the vasculature, central nervous system, and in endothelial cells. Intracellularly, the LOX isoforms are primarily found in the cytoplasm in their inactive form and upon activation, it will translocate to the nucleus or membrane (plasma, intracellular) (Czapski et al., 2016; Rådmark and Samuelsson, 2009). LOXs are involved in the production of leukotrienes, lipoxins, and hydroxy fatty acids; however, the biological roles for most LOX isoforms are not entirely known (Kuhn and Thiele, 1999). LOXs are capable of peroxidizing membrane lipids resulting in cellular structural changes (Rapoport and Schewe, 1986). The detailed mechanism of LOX is still not entirely known. However, there is a consensus regarding the radical nature of this reaction (Kuhn and Thiele, 1999; Czapski et al., 2016).

#### 4.4.1 Evidence of the effects of the exposome on LOX

Cytokines can regulate the expression of arachidonate lipoxygenase (*ALOX*) genes, with Th2 cytokines increasing *ALOX15* expression (Mashima and Okuyama, 2015). Human patients challenged with aeroallergens (i.e., birch, grass, house dust mite) and diesel exhaust also demonstrated an increase in the expression of *ALOX15* in the airway epithelium (Li et al., 2024). External exposures like calcium can regulate the expression of *ALOX5*, while acrolein, an environmental exposure released from burning tobacco, wood, plastics, gasoline, paraffin wax, and some cooking fats and oils, can increase the expression of 5-LOX via EGFR-dependent activation of the ERK pathway (Kim et al., 2010). Meanwhile zileuton is a pharmaceutically available 5-LOX inhibitor used to control asthma (Mashima and Okuyama, 2015). Phenolic antioxidants (i.e., nordihydroguaiaretic acid and caffeic acid) can also inhibit various LOX isoforms (Mashima and Okuyama, 2015).

#### 4.4.2 Evidence of the effects of LOX on aging and senescence

In general, LOX generates lipid hydroperoxides from PUFAs. These hydroperoxides can interact with metal ions to decompose into other free radicals, resulting in oxidative stress, which accelerates aging via increased cell death and cellular senescence (Mashima and Okuyama, 2015). *ALOX12* gene polymorphisms are associated with age-related diseases such as cancers (i.e., esophageal squamous cell, colorectal, and breast), neurological disorders, hypertension, and bone mineral density while *ALOXE3* and *ALOX12B* mutations together are associated with various skin disorders (Mashima and Okuyama, 2015). *ALOX5* is negatively associated with neurological disorders, as 5-LOX is upregulated in neuronal tissue during AD. Furthermore, treatment with the 5-LOX inhibitor zileuton in an AD mouse model demonstrated a rescuing effect from AD-like phenotype. Specifically, zileuton improved memory impairment, reduced Aβ plaques via decreased expression of γ-secretase complex, and reduced tau hyperphosphorylation via decreased expression of the binding activators (p35, p25) of the cgk-5 kinase pathway partially responsible for tau hyperphosphorylation (Chu et al., 2013). Meanwhile, an aging mouse model deficient in 12- and 15-LOX found that deficiency in 12/15-LOX leads to increased spleen mass and disruption to spleen microarchitecture, cardiac dysfunction and inflammation, and diminished essential immune cell populations and their phenotypes, overall contributing to age-related inner-organ inflammation (Kain et al., 2021).

The LA-derived 15-LOX metabolite, 13S-hydroperoxyoctadecaenoic acid, has been found to induce apoptosis in colon cancer cells (Shureiqi et al., 2000). In fibroblasts, oncogenic-, stress-, or radiation-induced senescence promoted secretion of leukotrienes and expression of enzymes responsible for leukotriene biosynthesis (Wiley et al., 2019). Following chemotherapy-induced senescence, renal injury was accelerated by cysteinyl leukotrienes (Rubinstein and Dvash, 2017). While leukotrienes are secreted from senescent cells, they appear to function in parallel to SASP factors (Hamsanathan and Gurkar, 2022).

### 4.5 Cyclooxygenase

Cyclooxygenases (COXs) consists of two isoforms in vertebrates (COX-1 and COX-2) that are responsible for the catalysis of PUFAs to prostaglandins, prostacyclin, thromboxanes, and hydroxy-PUFAs (Rouzer and Marnett, 2009; Rouzer and Marnett, 2003). Both COX isoforms are heterodimers, with each subunit of the dimer containing an epidermal growth domain, membrane-binding domain, and catalytic domain where the cyclooxygenase and peroxidase active sites are found on either side of the heme prosthetic group (Rouzer and Marnett, 2009). COX-1 is continuously expressed and is found in most tissues, while COX-2 expression is inducible (Rouzer and Marnett, 2003). COX-1 and COX-2 are membrane-bound proteins found in the nuclear membrane and in the lumen of the ER (Spencer et al., 1998). A third cyclooxygenase, COX-3, has also been identified as it results from a splice variant of the COX-1 gene. However, this enzyme is not functional in humans (Chandrasekharan et al., 2002). The catalysis of COX occurs in a similar manner to that of nonenzymatic peroxidation. However, COX restricts the location of hydrogen abstraction and will dictate reaction stereochemistry (Rouzer and Marnett, 2009; Rouzer and Marnett, 2003; van der Donk et al., 2002).

#### 4.5.1 Evidence of the effects of the exposome on COX

Both isoforms of COX are inhibited by NSAIDs; but COX-2 can be induced by a variety of environmental factors such as bacterial products, pro-inflammatory cytokines, UV light, cigarette smoke, diet, heavy metals, and xenobiotics (Kim et al., 2016). In endometriosis, upregulation of COX-2 by estrogen, hypoxia, proinflammatory cytokines, environmental pollutants, metabolites and metabolic enzymes, and platelets increases COX-2-derived prostaglandin E2 (PGE2) which can act in a feedback loop for enhanced inflammation (Lai et al., 2019). One study also found that COX-2 and its subsequent metabolite PGE2 are affected during temperature regulation in response to external temperatures (Esh et al., 2021). Dysregulated COX activity due to environmental exposures can contribute to several chronic conditions such as atherosclerosis, arthritis, cancer, diabetes, inflammation, neurodegenerative diseases, and osteoporosis as well as multidrug resistance (Kim et al., 2016; Lai et al., 2019).

#### 4.5.2 Evidence of the effects of COX on aging and senescence

Increased activity of COX-2 is associated with aging, and expression of COX-2 was upregulated in multiple organs in both aged rodent models and humans (Hamsanathan and Gurkar, 2022; Kim et al., 2016). COX-2 expression has also been reported to be increased in many age-related diseases (Kim et al., 2016). A transgenic mouse model with an inducible expression of COX-2 found that this induced expression resulted in several premature aging-related phenotypes and a significantly reduced lifespan, while lung fibroblasts from this model exhibited increased expression of SA-β-gal (Kim et al., 2016). In human lung fibroblasts an upregulation of COX-2 expression and subsequent production of PGE2 was observed in normal and stress-induced senescence, while arachidonic acid supplementation further enhanced this observation and accelerated the incidence of key senescent features (Dagouassat et al., 2013). Senescent cells were shown to produce and accumulate multiple prostaglandin J2 metabolites that can promote senescence arrest and SASP production along with other prostaglandin D2 metabolites (Wiley et al., 2021).

## 5 Conclusion

There is a current lack of understanding about the intersection of the exposome in geroscience, resulting in many unknowns regarding the mechanisms controlling healthy aging. Previous research has demonstrated epigenetic changes due to environmental exposures (Miller and Jones, 2014), with researchers suggesting that environmental exposures are more impactful than genetic factors in human diseases such as cancer and degenerative diseases (Rappaport, 2011). Some studies have demonstrated that immigrant populations adopt the disease patterns of their host countries (KATO et al., 1973), and that there are regional distinctions in disease rates of the same disease throughout the separate United States populations (KATO et al., 1973). While there is a higher familial risk of certain types of cancer in twin studies, genetics alone is not sufficient to adequately characterize disease risk (Mucci et al., 2016). However, some studies have also examined the specific relationship between environmental exposures and specific mutations in tumors, highlighting the joint contribution of genome and exposome in cancer (Willett, 2002). Due to the large spread of environmental exposures that may contribute to a decline in health and/or involvement in human disease, it is not currently feasible to quantify and examine all these factors. Thus, much of the research in the field of exposure science examines exposures (toxicants) with previously known biologically active roles, such as reactive electrophiles, metabolites, metals, endocrine disruptors, persistent organic compounds, and modulators of immune responses (Rappaport and Smith, 2010). As discussed throughout this review, exposure to many of these substances can affect aging in terms of their involvement in age-related diseases and their contribution to oxidative stress, cell death, and senescence.

### 5.1 Potential developments in the field

As the exposome encompasses such a large range of exposures and so many of these exposures can interact, a huge knowledge gap remains about how these exposures can co-function in affecting oxylipin metabolism *in vivo* and in aging-related effects. However, due to the extent of these exposures, it is not feasible to individually test all of these and in combination with each other. Thus, high throughput screening is needed to examine the interaction of the exposome on the oxylipin profile at a large scale, increasing our knowledge of the human relevance of these exposures. Utilizing high throughput screening, additional exposures not detailed within this review should also be examined, such as per- and polyfluoroalkyl substances (PFAS), lifestyle factors (i.e., alcohol, tobacco, caffeine), pharmaceuticals, and other environmental and occupational contaminants for their effects on both oxylipin profile and healthy aging.
