# Supplementary material for: Effects of molecular interactions between the exposome and oxylipin metabolism on healthspan
Source: Front Physiol. 2025 Jul 1;16:1584195. doi: 10.3389/fphys.2025.1584195 (PMC12259568; doi:10.3389/fphys.2025.1584195)

Figure 1. Vitamin E Metabolites. VitE can be found in two forms: tocopherols and tocotrienols, which can be designated as alpha (α), beta (β), gamma (γ) or delta (δ) based on the number and position of methyl group(s) on the R_1_ and R_2_ side chains. Created with BioRender.com.


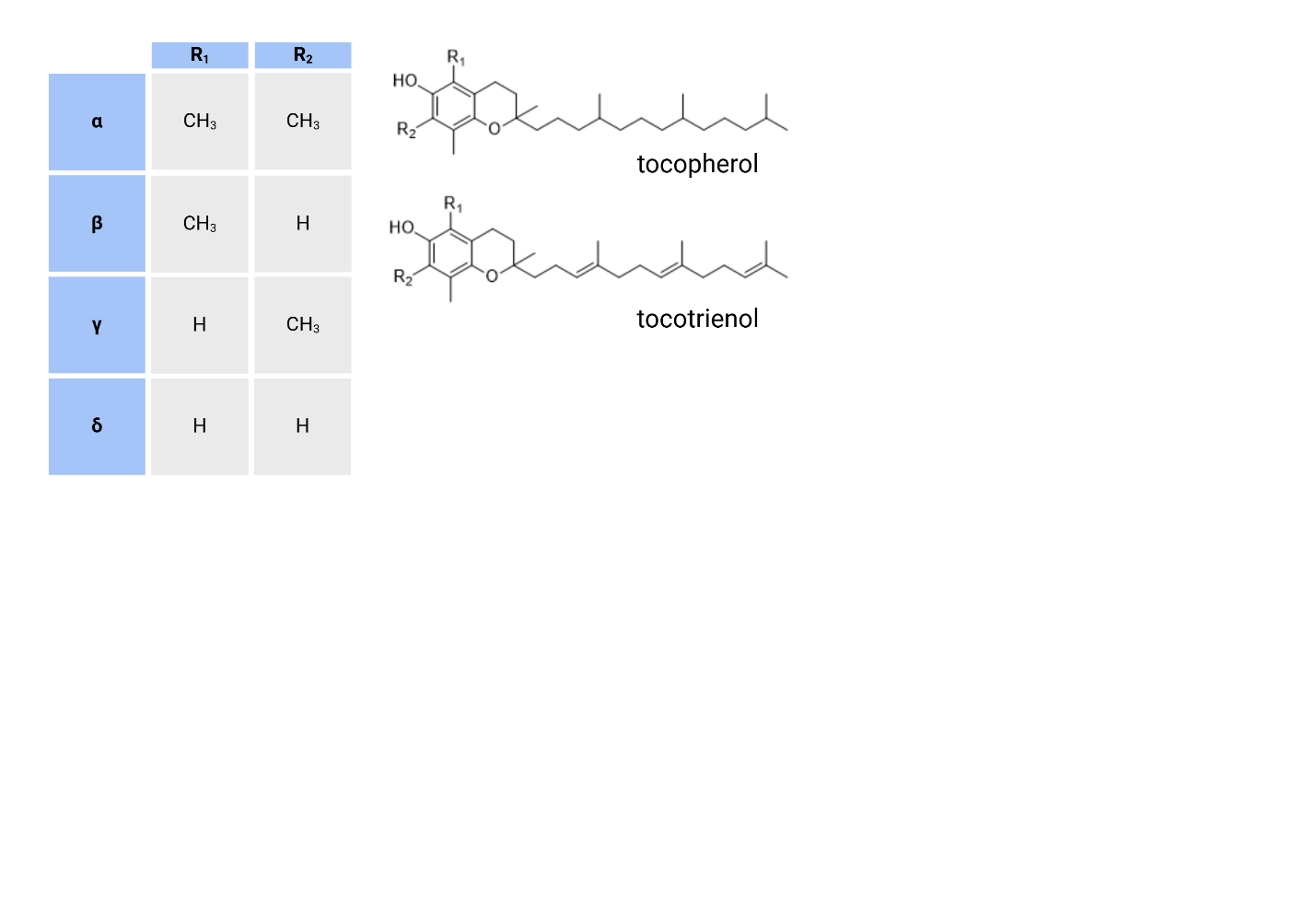


Figure 2. Vitamin K Metabolism. VitK can be found in three forms – VitK1 and VitK2 naturally, and synthetic VitK3. The VitK cycle can continuously regenerate VitK, VitK hydroquinone, and VitK epoxide in which this cycle can active VitK-dependent proteins (VKDP). DTT: dithiothreitol, DTTH2: disulfide-bonded 6-membered ring. Created With BioRender.com.


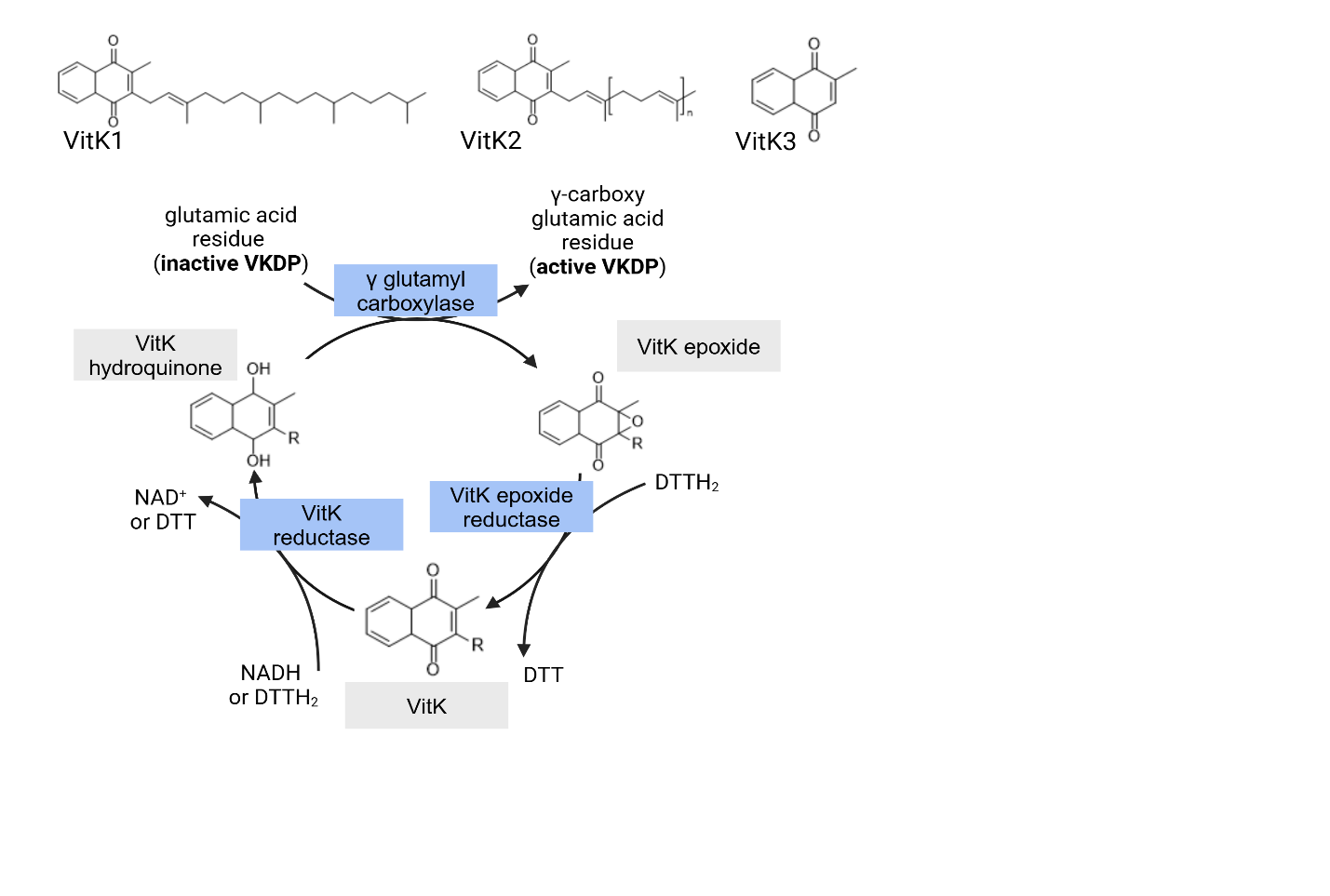


Figure 3. PUFA Biosynthesis Pathway. Dietary intake of LA and ALA can be converted into their respective downstream PUFAs. LA: linoleic acid, GLA: γ-linolenic acid, DGLA: dihomo- γ-linolenic acid, AA: arachidonic acid, ADA: adrenic acid, DPA6: docosapentaenoic acid, TTA: tetracosatetraenoic acid, TPA6, tetracosapentenoic acid (ω-6), ALA: α-linolenic acid, STA: stearidonic acid, ETA: eicosatrienoic acid, EPA: eicosapentaenoic acid, DPA3: docosapentaenoic acid, DHA: docosahexaenoic acid, TPA3: tetracosapentenoic acid (ω-3), THA: tetracosahexaenoic acid. Created with BioRender.com.


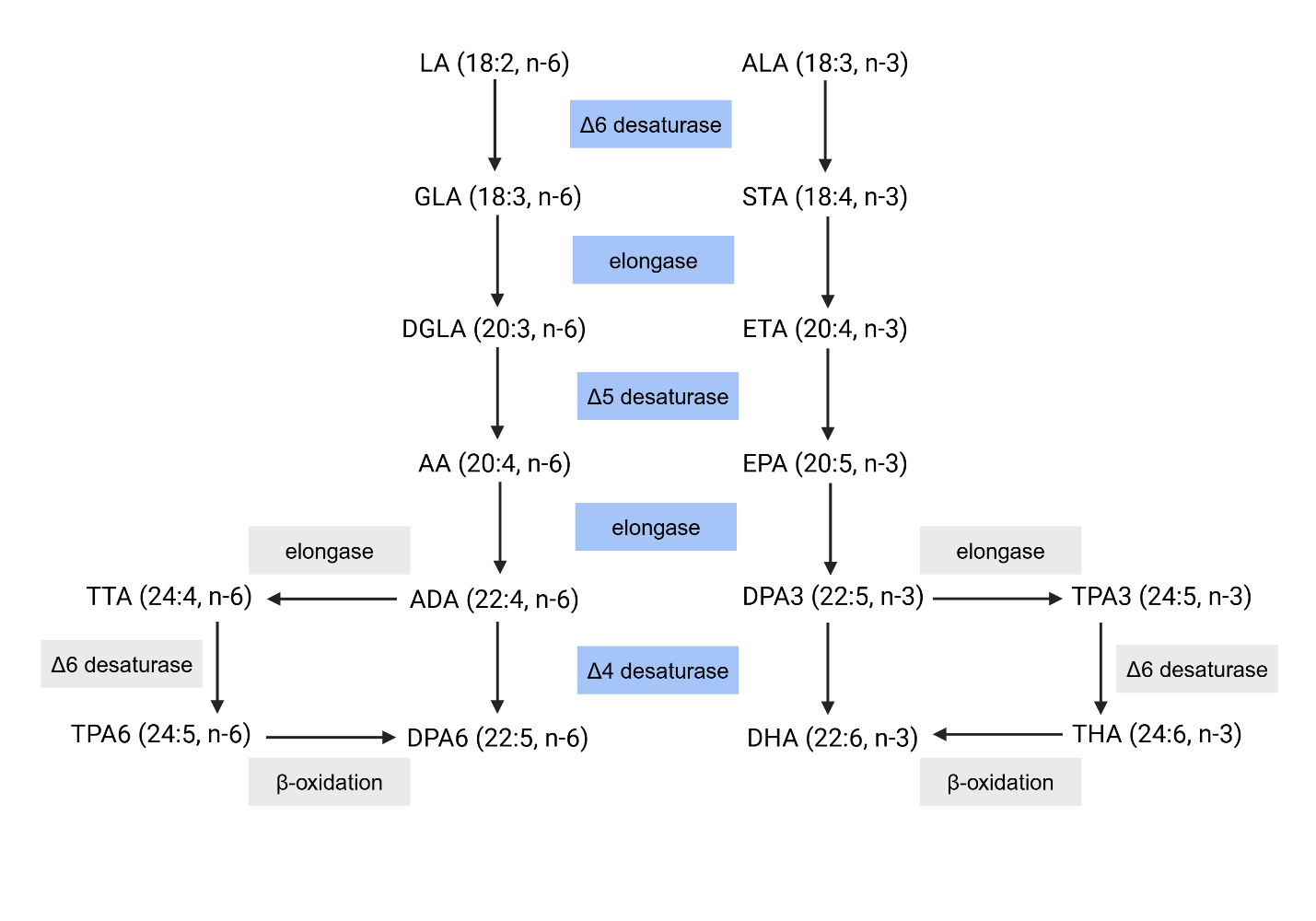


Figure 4. PUFA Enzymatic Metabolism. A range of PUFA metabolites can be produced via CYP-sEH (cytochrome P450-soluble epoxide hydrolase), COX (cyclooxygenase), or LOX (lipoxygenase)-mediated reactions. AA: arachidonic acid, EET: epoxyeicosatrienoic acid, DHET: dihydroxyeicosatrienoic acid, HETE: hydroxyeicosatetraenoic acid, PGE2: prostaglandin E2. Created with BioRender.com.


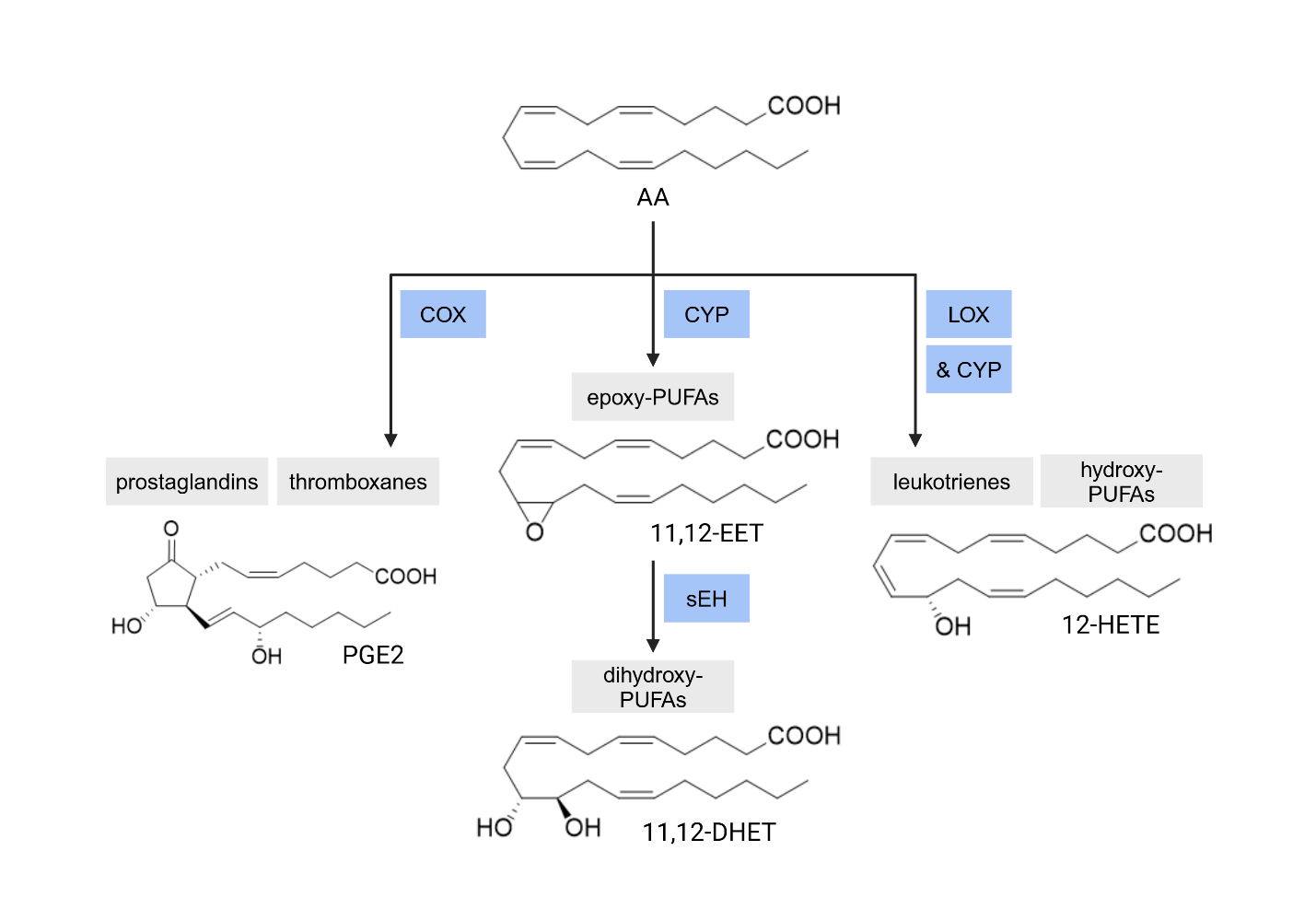

Supplement: Supplementary file 1 [file DataSheet1.docx]
